# Diagnostic Techniques for Posterior Cutaneous Nerve Entrapment Syndrome

**DOI:** 10.7759/cureus.74639

**Published:** 2024-11-27

**Authors:** Takashi Watari

**Affiliations:** 1 Integrated Clinical Education Center, Kyoto University Hospital, Kyoto, JPN; 2 General Medicine Center, Shimane University Hospital, Izumo, JPN

**Keywords:** anterior cutaneous nerve entrapment syndrome, lateral cutaneous nerve entrapment syndrome, physical diagnosis, posterior cutaneous nerve entrapment syndrome, video, video lecture capture

## Abstract

This case report highlights the diagnostic process for posterior cutaneous nerve entrapment syndrome (POCNES) in an older adult female patient, with an emphasis on using video demonstrations to guide clinicians. POCNES should be diagnosed based on a combination of specific clinical features, including localized back pain accompanied by pinpoint tenderness lateral to the spinous process, cutaneous sensory abnormalities over the area of pain, severe pain response to pressure on the tender point, and normal clinical and imaging findings. This case demonstrates how these criteria were identified via a thorough physical examination captured on video to provide a visual guide for clinicians. The use of a lidocaine injection at the tender point confirmed the diagnosis, offering immediate relief. The video in this report serves as a practical tool for clinicians to accurately diagnose POCNES, particularly when standard imaging and tests remain inconclusive.

## Introduction

Posterior cutaneous nerve entrapment syndrome (POCNES) is often underdiagnosed. Recently, anatomically, POCNES has been suggested to be a neuropathic pain condition similar to anterior cutaneous nerve entrapment syndrome (ACNES) and lateral cutaneous nerve entrapment syndrome (LACNES) [1-3]. However, established diagnostic criteria for diagnosing POCNES have not been determined, and it relies on the clinical reasoning skills of clinicians [1,2]. A thorough record of pain history and meticulous physical examination are essential for diagnosing POCNES. However, there is a lack of documented literature, including textbooks, case reports, and papers, on how to conduct these examinations [2]. Therefore, we aimed to present case video using actual patient examinations to introduce diagnostic methods and raise awareness of examination techniques for this condition among clinicians.

## Case presentation

A female patient in her 80s, who is independent in activities of daily living and uses a walking cane, was transported to the emergency department with severe back pain that she had never experienced in her life. The pain appeared three days prior when she bent down to pick something up from the floor. Initially, she visited the orthopedic department of the same hospital and was prescribed radiography and ultrasound examinations, which revealed no abnormalities. Hence, the condition was diagnosed as muscle pain. The pain showed a worsening trend, continuing as a sharp back pain. The patient's pain worsened with movement, preventing her from turning over in bed, causing her to suffer during sleep, prompting her husband to call for an emergency. Upon emergency transport, the patient was unable to perform rotational and flexion-extension movements of the torso, maintain sitting and standing postures, or walk due to the excruciating pain. Every movement triggered a severe electric shock-like pain (NRS=10/10). However, the patient expressed that the pain subsided to a Numerical Rating Scale of 3/10 when at rest.

**Figure 1:**
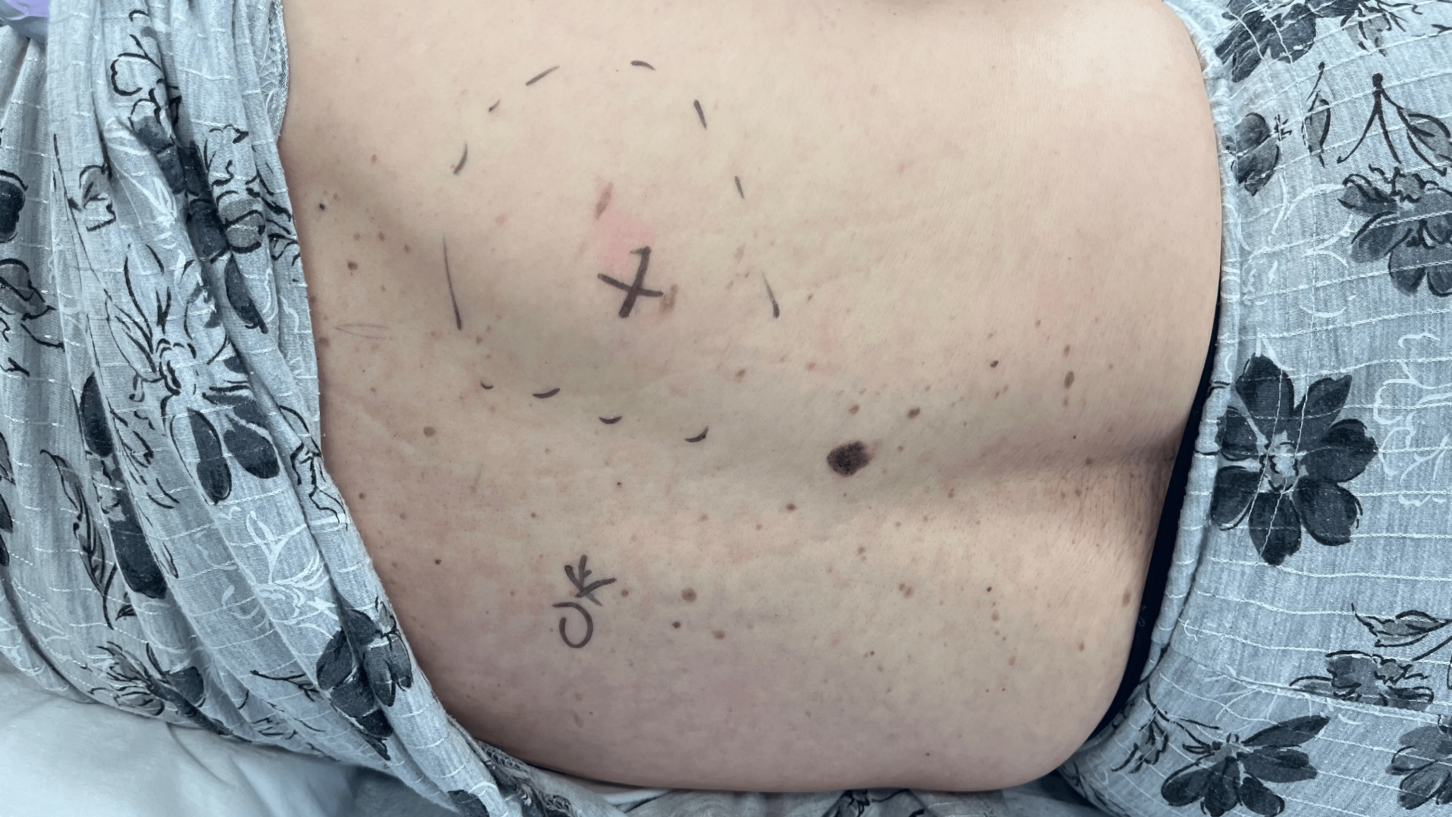
Identification of pain areas and the strongest pain points. In order to pinpoint the exact location of pain in the patient's posterior cutaneous nerve area, the patient is asked to confirm the degree of pain and abnormal sensations using pinch signs, marking each one accordingly.

She was conscious at admission, her vital signs were normal, and there was no difference in blood pressure between both her upper arms. Visual inspection of the back revealed no abnormalities, including rashes. However, palpation revealed a strong tender point in a narrow area (approximately 2cm x 2cm, indicated by an "X" mark) approximately 5cm to the right of the midline at the T6 vertebra (Figure 1) with a numerical rating scale (NRS) of 10/10. Even light physical contact induces pain, while pinching elicits severe pain (positive pinch sign) [4].

She described the pain as tingling and like being pinched with pliers. Furthermore, she complained of severe pain even with the friction of an alcohol swab and reported abnormal sensations such as numbness and tingling around the area. Fingers were used to carefully identify and mark the pain and abnormal sensation areas. Based on the markings, it was determined that the pain and abnormal sensation areas matched the posterior cutaneous nerve region. A local injection of 5 mL of 1% lidocaine was administered at the most tender point marked with an "X." Within three minutes after the injection, her back pain and hyperesthesia completely disappeared, confirming the diagnosis of POCNES (Video 1).

**Video 1:** Diagnostic techniques for posterior cutaneous nerve entrapment syndrome. A detailed pain history assessment, including the timeline, aggravating factors, and pain intensity, was conducted to determine if the pain is similar to that felt during an electric shock and identify the presence of abnormal sensations (such as hypoesthesia or hyperesthesia). During this process, the exact location of the most intense pain, as well as the precise areas of abnormal sensation, were marked. Furthermore, whether light pinching induces pain or abnormal sensations was checked. Thereafter, 5 mL of 1% lidocaine was injected at the most tender point and observed for a few minutes. Finally, the effect of the treatment was evaluated; improvement indicates impairment in the posterior cutaneous nerve area, allowing for a more precise diagnosis. In this case, the abnormal sensations in the marked areas significantly improved, and the patient was able to walk again after the injection.

Subsequently, a computed tomography was performed to rule out compression fractures or nerve root impairments and thoracic wall abnormality, which are rare causes of POCNES, but no abnormalities were found. After the initial treatment, the residual mild pain in the deeper area disappeared, and 15 minutes later, the patient was able to walk with a cane, as before the incident. As the pain recurred, she visited a pain clinic, where the pain improved without surgical intervention.

## Discussion

Anatomically and pathophysiologically, POCNES is characterized by distinctive pain caused by compression, entrapment, or physical impairment of the posterior cutaneous nerve [1,2]. Owing to its nature, blood tests and imaging studies are generally not useful in ruling out the diagnosis of POCNES [3]. Therefore, careful attention to the patient's pain is essential for the accurate diagnosis of POCNES. This includes integrating information about the nature and intensity of pain, its duration and course, precise location, positions that exacerbate or relieve pain, and the presence of hyperesthesia or hypoesthesia while maintaining neuroanatomical awareness. Accurate identification of the nerve impairment area is crucial for a precise diagnosis, which involves marking painful areas with a pen and identifying the point of maximum tenderness. Without this, a diagnosis is nearly impossible [3]. Clinically, the most definitive findings for the diagnosis of POCNES are a positive pinch test and improvement following lidocaine injection at the point of maximum tenderness [3,4]. According to a review by Otsuka et al., the pinch test is positive in more than 80% of cases [2]. The pinch test, as demonstrated in the video, involves gentle pinching and pulling the area where the patient reported pain. If a patient experiences worsening pain or abnormal sensations, even at a pressure level that would not typically cause pain, the test is considered positive [4]. Additionally, lidocaine injections are effective in more than 75% of patients, significantly increasing the likelihood of a diagnosis [2]. However, the injection must be administered close to the site of nerve entrapment for optimal effectiveness, and its efficacy may be reduced if the injection is incorrectly positioned or if the medication does not reach the affected area. Differential diagnosis includes the following (Table 1).

**Table 1:** Differential diagnosis of posterior cutaneous nerve entrapment syndrome.

Differential diagnosis
Facet joint arthropathy/arthritis
Rib abnormalities
Radiculopathy (traumatic, diabetic)
Scar tissue
Herniated disc
Myofascial pain syndrome
Postherpetic neuralgia
Thoracic wall abnormality (hematoma, endometriosis, tumor, tear)
Slipping rib syndrome
Neurofibroma
Bornholm disease
Schwannoma

Regarding the site of onset, a review of POCNES indicated that 68.9% of cases were most commonly affected at the T11-T12 vertebral levels, with no significant laterality observed, although there were two bilateral cases [2]. Many currently reported cases of POCNES involve younger individuals with a median age of 26 years [2]. Additionally, 87.5% of the patients were women, although the reason for this sex predominance is unknown. As for age, these case reports did not include older adult patients with surgical scars or a history of spinal surgery, which could change the common age range in future studies since age is unlikely to be a significant factor, given the pathophysiology and anatomical principles of POCNES [3]. Although facet joint arthropathy or arthritis is a common differential diagnosis due to the similarity in pain characteristics, more urgent and severe differential diagnoses should also be systematically evaluated. Interestingly, 37.5% of patients had a history of anterior cutaneous nerve entrapment syndrome (ACNES) [2], suggesting a potential common cause of ACNES and lateral cutaneous nerve entrapment syndrome (LACNES), considering their anatomical and pathophysiological similarities. ACNES has been reported to appear in the same nerve distribution area after the diagnosis and treatment of LACNES [5].

Finally, the average delay in diagnosing POCNES was 22 months (range: 5-48 months), with 69% of the patients eventually undergoing neurectomy, although some pain remained [2]. The relatively long time to diagnosis in the literature can be attributed to the dependence on the physician's knowledge and experience with POCNES and the detailed examination techniques mentioned above, which often results in underdiagnosis [1,2,3]. In this case, the time to diagnosis (TTD) was very short because the following diagnostic criteria were considered while examining the patient based on the illness script of electric shock-like back pain: The time needed for diagnosis was not due to the inherent pathophysiology or anatomical factors of the disease (such as the time needed to complete the illness script or the appearance of typical symptoms). Therefore, it may be advisable for physicians to exclude the time taken for diagnosis in the POCNES diagnostic classification. In conclusion, the following criteria should be widely recognized by physicians for diagnosis:

POCNES is defined by the following criteria: localized back pain; a circumscriptive area of tenderness lateral to the spinous process covering a small and predictable point of maximal pain; a larger area of cutaneous somatosensory abnormalities (such as hypoesthesia, hyperesthesia, and/or altered cold perception) overlying this maximal pain point; local pressure on the tender point resulting in a predictable, severe pain response; and normal clinical and imaging findings.

## Conclusions

Accurate diagnosis of POCNES requires meticulous attention to the patient's pain characteristics, including its nature, intensity, location, and associated sensory abnormalities. A thorough physical examination, guided by a strong understanding of neuroanatomy, is essential to identify the specific regions of nerve involvement accurately. While blood tests and imaging studies are generally unhelpful for diagnosing POCNES, clinical expertise plays a pivotal role. Key diagnostic tools include detailed history-taking, physical examination, and targeted tests such as the pinch test and lidocaine injection at the most tender point. 
